# Reduced Sensitivity to Sooner Reward During Intertemporal Decision-Making Following Insula Damage in Humans

**DOI:** 10.3389/fnbeh.2015.00367

**Published:** 2016-01-12

**Authors:** Manuela Sellitto, Elisa Ciaramelli, Flavia Mattioli, Giuseppe di Pellegrino

**Affiliations:** ^1^Dipartimento di Psicologia, Università di BolognaBologna, Italy; ^2^Centro Studi e Ricerche in Neuroscienze Cognitive, Polo Scientifico-Didattico di CesenaCesena, Italy; ^3^Riabilitazione Neuropsicologica, Spedali Civili di BresciaBrescia, Italy

**Keywords:** emotion, insular cortex, limbic system, reward, temporal discounting, visceral factors

## Abstract

During intertemporal choice, humans tend to prefer small-sooner rewards over larger-delayed rewards, reflecting temporal discounting (TD) of delayed outcomes. Functional neuroimaging (fMRI) evidence has implicated the insular cortex in time-sensitive decisions, yet it is not clear whether activity in this brain region is crucial for, or merely associated with, TD behavior. Here, patients with damage to the insula (Insular patients), control patients with lesions outside the insula, and healthy individuals chose between smaller-sooner and larger-later monetary rewards. Insular patients were less sensitive to sooner rewards than were the control groups, exhibiting reduced TD. A Voxel-based Lesion-Symptom Mapping (VLSM) analysis confirmed a statistically significant association between insular damage and reduced TD. These results indicate that the insular cortex is crucial for intertemporal choice. We suggest that he insula may be necessary to anticipate the bodily/emotional effects of receiving rewards at different delays, influencing the computation of their incentive value. Devoid of such input, insular patients’ choices would be governed by a heuristic of quantity, allowing patients to wait for larger options.

## Introduction

Intertemporal choice requires weighting sooner temptations against long-term, larger gratifications. In the laboratory, these situations may be modeled by manipulating the time at which rewards are delivered. For example, a subject might choose between $5 now and $15 in 1 week. Humans, as well as other animals, tend to prefer sooner-smaller over later-larger rewards (e.g., Ainslie, 1974). The decrease in subjective value of a reward as the delay until its receipt increases is called temporal discounting (TD; Ainslie, 1975).

Given the pervasive problems associated with short-sighted decision-making, such as addiction and impulsivity (Bickel et al., 2007), there is increasing interest in specifying the neural underpinnings of TD. Previous evidence suggests that intertemporal choice is governed by a valuation process estimating the incentive value of different options, and a control process that exerts top-down modulation over valuation, pursuing long-term goals (Peters and Büchel, 2011). The medial orbitofrontal cortex (mOFC) and ventral striatum, and the dorsolateral prefrontal cortex, are core areas of the valuation and the control network, respectively (Rangel et al., 2008; Figner et al., 2010; Sellitto et al., 2011).

We hypothesize that another region crucially involved in TD may be the insula. It has long been known that the insula is implicated in decision-making. Several functional neuroimaging (fMRI) studies have implicated the insula in choice about money, drug, and other goods (e.g., Knutson et al., 2000; Guillem et al., 2010; Tusche et al., 2010), especially during decisions involving uncertainty (Huettel et al., 2006) and risk (Kuhnen and Knutson, 2005). Crucially, activity in the insula has been recently found to modulate according to the time of availability of edible and monetary outcomes (Tanaka et al., 2004; McClure et al., 2007; Wittmann et al., 2007, 2010; Claus et al., 2011; Liu and Feng, 2012; Luo et al., 2012), suggesting that this brain region may be implicated in intertemporal choice. fMRI evidence, however, have not univocally associated insula activity with choice of either delayed (Wittmann et al., 2007; Claus et al., 2011; Kayser et al., 2012; Liu and Feng, 2012; Luo et al., 2012) or immediate options (Tanaka et al., 2004; McClure et al., 2007; Wittmann et al., 2010), and therefore its role in TD behavior is unclear. Moreover, fMRI cannot clarify whether activity in a brain region is imperative for, or instead spuriously associated with, a behavior of interest (e.g., Rorden and Karnath, 2004; Poldrack and Farah, 2015). To overcome this limitation, and provide causal evidence about the role insula activity plays in intertemporal decision-making, we used a lesion approach: if activity in the insula is crucial for intertemporal choice, then patients with lesion to this brain region should show abnormal TD behavior.

One plausible hypothesis is that the insula may influence the probability of selecting sooner vs. later options based on individuals’ physiological state (Volkow and Baler, 2015). The insula has been associated with the conscious representation of bodily states, and the anticipation of the bodily effects of emotional events (Rolls, 1999; Damasio et al., 2000; Craig, 2009). By signaling the current needs of the body, such inputs may influence the valuation of different goods, and generate conscious urges capable of driving behavior (e.g., Craig, 2009; Naqvi and Bechara, 2009). During intertemporal choice, bodily signals may convey the urge to obtain a reward soon, overwhelming attempts to implement far-sighted decisions (Loewenstein, 1996; Camerer et al., 2004). For this reason, we predicted that lesions to the insula would decrease the appetitive value of sooner rewards, leading to reduced TD.

In order to examine whether insula activity is crucial for intertemporal choice, and begin to shed light on its specific role for TD, patients with lesion to the insula (Insular patients), control patients with lesions outside the insula, and healthy participants chose between smaller-sooner rewards and larger-later rewards (Experiment 1). In line with the hypotheses, Insular patients showed reduced TD, and in a corollary investigation (Experiment 2), reduced arousal for positive stimuli.

## Experiment 1

### Materials and Methods

#### Participants

Participants included 25 patients with brain damage and 64 healthy individuals (see Table 1 for demographic and clinical information). Patients were recruited at the Centre for Studies and Research in Cognitive Neuroscience, Cesena, Italy, and at the Spedali Civili of Brescia, Italy. They were selected on the basis of the location of their lesion evident on magnetic resonance imaging (MRI) or computerized tomography (CT) scans, and divided into two groups based on their lesion location.

**Table 1:** **Participant groups’ demographic and clinical data [mean (standard deviation)]**.

	Sex (M/F)	Age (years)	Education (years)	BDI-II	MMSE	DS	CRM	Lesion volume (cc.)
Insular (*n* = 12)	7/5	60.6 (14)	10.5 (4.9)	10.6 (8.7)	25.1 (2)	5.0 (1.2)	28.8 (4.6)	33.3 (23.5)
Non-insular (*n* = 13)	6/7	58.9 (12.7)	10.1 (3.8)	9.9 (7.1)	25.6 (1.6)	5.8 (1.3)	25.5 (3.1)	25.4 (11.7)
HC (*n* = 64)	33/31	54.2 (14.5)	11.4 (4)	–	–	–	–	–

*Insular, patients with lesions in the insular cortex; Non-insular, patients with lesions outside the insula; HC, healthy controls; F, female; M, male; BDI-II, Beck Depression Inventory Scale; MMSE, Mini Mental State Examination (corrected score); DS, digit span forward (corrected score); CRM, Colored Raven Matrices (corrected score)*.

**Figure 1:**
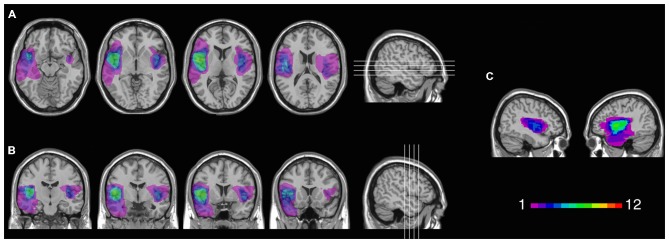
**Location and overlap of brain lesions in Insular patients.** The image shows the lesions of the Insular patients projected on the same four axial slices **(A)** and on the same four coronal slices **(B)** of the standard Montreal Neurological Institute (MNI) brain. In each slice, the left hemisphere is on the left side. The level of axial and coronal slices has been marked by white lines on the sagittal view of the brain. Sagittal views of the MNI brain show the degree of lesion coverage for the right- and left-lesioned Insular patients; the color bar indicates the number of overlapping lesions **(C)**.

**Figure 2:**
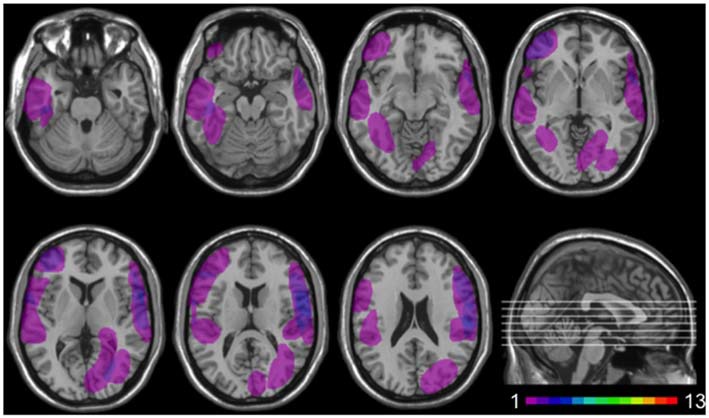
**Location and overlap of brain lesions in Non-insular patients.** The image shows the lesions of the Non-insular patients projected on the same seven axial slices. In each slice, the left hemisphere is on the left side. The level of the axial slices has been marked by white lines on the sagittal view of the brain. The color bar indicates the number of overlapping lesions in the axial slices.

Twelve patients (5 females) had lesions involving the insular cortex, hidden in the lateral sulcus, covered by frontal, temporal, and parietal opercula, as well as surrounding gray and white matter (see Figure 1). Since lesions predominantly involved the insular cortex (see “Results” Section), we henceforth refer to this group as “Insular patients”. Lesions were caused by an ischemic or hemorrhagic stroke of the middle cerebral artery (MCA) and were unilateral in all cases (left hemisphere: 8 cases, right hemisphere: 4 cases). Thirteen patients (7 females) had brain damage that spared the insular cortex in both hemispheres (see Figure 2). We henceforth refer to this group as “Non-insular patients”. Lesions were caused by ischemic or hemorrhagic stroke (9 cases) or tumor resection (4 cases), were unilateral in all cases (left hemisphere: 7 cases, right hemisphere: 6 cases), and mainly involved the occipital cortex, the temporal cortex, and the superior frontal cortex. In no case did patients’ lesions involve the mOFC (see Sellitto et al., 2010). There was no significant difference in lesion volume between Insular patients and Non-insular patients (33.33 vs. 25.36 cc.; *p* = 0.29).

All patients were in the chronic phase of recovery (at least 12 months post onset), were not receiving psychoactive drugs, and had no other diagnosis likely to affect cognition or interfere with the participation in the study (e.g., significant psychiatric disease, alcohol abuse, history of cerebrovascular disease). Patients’ general cognitive functioning was generally preserved, as indicated by the scores they obtained in the Mini-Mental State Examination (MMSE; Folstein et al., 1975), the digit span forward test (DS), and the Colored Raven Matrices (CRM), which were within the normal range in all cases (Spinnler and Tognoni, 1987; see Table 1). In addition, left-damaged patients had no aphasia documented, and right-damaged patients had no hemispatial neglect documented.

The healthy control group comprised 64 individuals (31 females) matched to the patients on demographic ground, including mean age, gender, and level of education. Control participants were not taking psychoactive drugs, and were free of current or past psychiatric or neurological illness as determined by history.

All participants gave informed consent, according to the Declaration of Helsinki (International Committee of Medical Journal Editors, 1991) and the Ethical Committee of the Department of Psychology, University of Bologna.

#### Lesion Analysis

Patients’ individual lesions, derived from the most recent clinical MRI or CT images, were manually drawn by a neurologist (blind to patients’ performance) directly on each slice of a T1-weighted template MRI scan from the Montreal Neurological Institute (MNI^1^; see also Karnath et al., 2004; Moro et al., 2008; Tsuchida and Fellows, 2012). This template is approximately oriented to match Talairach space (Talairach and Tournoux, 1988) and is distributed with MRIcron (Rorden and Brett, 2000). The standard template provides various anatomical landmarks to help experts plot the size and localization of the lesion using structural features such as sulci and gyri as guides. This manual procedure combines segmentation (identification of lesion boundaries) and registration (to a standard template) into a single step, with no additional transformation required (Kimberg et al., 2007). Manual segmentation/registration procedures have the limit to rely greatly on anatomical expertise, and to be subjective in nature. On the other hand, they circumvent problems frequently encountered by automated normalization procedures, such as warping scans from individuals with brain injury, which may be affected by structural distortions related to the lesion and not easily compensated for (e.g., ventricular enlargement, large regions of atypical voxel intensity values, etc), and combining subjects scanned with different imaging modalities (e.g., MRI vs. CT; see Fiez et al., 2000; Kimberg et al., 2007). MRIcron softwares were used to estimate lesion volumes (in cc.) and to generate lesion overlap images.

Figure 1 shows the extent and overlap of brain lesions in Insular patients. As is evident, although all patients had damage that included the insula, areas adjacent to the insula that are within the MCA blood supply territories were damaged to some degree as well. This included parts of the somatosensory cortex, the basal ganglia (e.g., caudate, putamen), the temporal lobe (especially the superior portions), the dorsolateral and/or ventrolateral prefrontal cortex, the inferior parietal lobule, the occipito-parietal junction, and the dorsomedial sectors of the occipital lobe. Lesions of Insular patients overlapped maximally in Brodmann Area (BA 20) (*M* = 7.0 cc., *SD* = 11.0 cc.), BA 21 (*M* = 2.7 cc., *SD* = 5.5 cc.), BA 38 (*M* = 2.1 cc., *SD* = 2.7 cc.), and BA 22 (*M* = 1.3 cc., *SD* = 2.2 cc.), as well as not numbered areas (*M* = 18.1 cc., *SD* = 6.4 cc.). Since the insular cortex is part of the areas with no Brodmann label (e.g., Kurth et al., 2010), we calculated maximal overlap location also using the Automated Anatomical Labeling (AAL) template (Tzourio-Mazoyer et al., 2002). This included the left (*M* = 6.6 cc., *SD* = 2.7 cc.) and the right (*M* = 6.5 cc., *SD* = 1.3 cc.) insula, as well as the temporal lobe bilaterally [temporal pole (*M* = 1.9 cc., *SD* = 2.4 cc.); superior portions of the temporal lobe (*M* = 5.2 cc., *SD* = 3.3 cc.)].

#### Temporal Discounting Task

In a computerized TD task, participants chose between an amount of reward that could be received sooner and an amount of reward that could be received later. Two temporal conditions were included. In the now condition, participants made a series of choices between a smaller amount of money (in €) that could be received immediately (now), and 40 € that could be obtained after a variable delay. In the Not-now condition, choices involved a smaller amount of money that could be received in 60 days, and 40 € that could be delivered after a variable delay larger than 60 days, while maintaining the same temporal gaps between earlier and later rewards as in the Now condition. Thus, in the Now condition participants made five choices at each of six delays: 2, 14, 30, 90, 180, and 365 days, whereas in the Not-now condition the delays were 62, 74, 90, 150, 240, and 425 days (see also Kable and Glimcher, 2010).

Within each block of five choices, the amount of the sooner reward was adjusted based on the participant’s previous choice, using a staircase procedure that converged on the amount of the sooner reward that was equal, in subjective value, to the later reward. The first choice was between a later amount of 40 € and a sooner amount of 20 €. If the sooner reward was chosen, then the amount of the sooner reward was decreased on the next trial; if the later reward was chosen, then the amount of the sooner reward was increased on the next trial. The size of the adjustment in the sooner reward decreased with successive choices: the first adjustment was half of the difference between the sooner and the later reward, whereas for subsequent choices it was half of the previous adjustment (Myerson et al., 2003). This procedure was repeated until the subject had made five choices at one specific delay, after which the subject began a new series of choices at another delay/temporal condition. For each trial in a block, the sooner amount represented the best guess as to the subjective value of the later reward. Therefore, the sooner amount that would have been presented on the sixth trial of a delay block was taken as the estimate of the subjective value of the later reward at that delay.

Moreover, two control conditions were included. In one, subjects made five choices between 40 € and a smaller amount of money, both available immediately. In the other, participants made five choices between 40 € and a smaller amount of money, both available in 365 days. The amount of the smaller option of the two control conditions was adjusted based on the staircase procedure described above. Both patients and healthy subjects always chose the larger reward in the two control conditions, suggesting adequate comprehension of the task as well as adequate sensitivity to reward.

The blocks of choices pertaining to the two temporal and control conditions were interspersed, and the order of blocks of choices relative to different delays of both temporal conditions was randomized for each participant.

Participants did not receive the actual consequences of their choices, but instead made choices about hypothetical rewards. While using hypothetical rewards has both advantages and disadvantages, there is no evidence that hypothetical rewards are discounted differently from real rewards, either in terms of the degree, shape, and neural bases of TD (Johnson and Bickel, 2002; Bickel et al., 2009). Moreover, in a previous work, we confirmed our results on hypothetical rewards using real rewards (Sellitto et al., 2010).

#### Self-Report Depression Scale

Given that the insular cortex has been implicated in depression (e.g., Takahashi et al., 2010; Sprengelmeyer et al., 2011), patients in the present experiment were administered the Beck Depression Inventory-II (BDI-II; Beck et al., 1996; Ghisi et al., 2006), a 21-item self-report questionnaire evaluating the presence and the severity of several aspects of depression symptoms. The BDI-II assesses, on a 4-point Likert scale, two components of depression: the affective component (AC subscale, e.g., “I do not expect things to work out for me”), and the somatic component (SC subscale, e.g., “I have less energy than I used to have”; e.g., Steer et al., 1999). A total score between 0 and 13 indicates minimal depression, 14–19 indicates mild depression, 20–28 indicates moderate depression, and 29–63 indicates severe depression.

#### Procedure

Before the beginning of the experimental session, participants were told that, on each trial, two hypothetical amounts of money would appear on the screen. One could be received sooner, and one could be received later. They were informed that there were no correct or incorrect choices, and were required to indicate the option they preferred by pressing one of two buttons (Sellitto et al., 2010).

**Figure 3:**
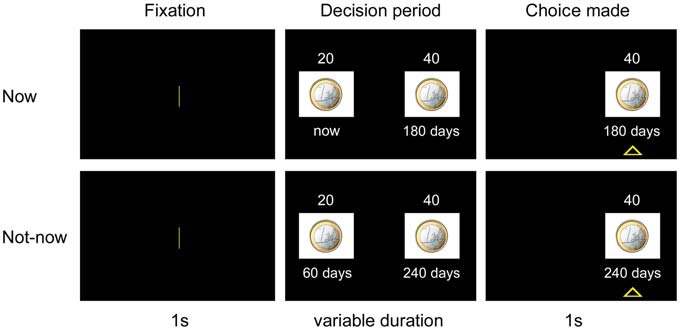
**Experimental paradigm.** In each trial, after a 1 s-fixation period, subjects chose between a small amount of reward delivered sooner and a larger amount of reward delivered after a longer delay. The preferred option remained highlighted for 1 s. The upper panel refers to a choice trial in the Now condition. The lower panel refers to a choice trial in the Not-now condition. See “Experiment 1” Section for a more detailed explanation of procedures.

Figure 3 illustrates the experimental paradigm. Each trial began with a 1 s fixation screen, followed by a screen depicting the two available options. The two options appeared on the left and right side of the screen, indicating the amount and the delay of delivery of the reward. After the participants made their decisions, the non-chosen option disappeared, whereas the preferred option remained on the screen for 1 s with a triangle underneath it. The inter-trial interval was 1.5 s.

Once the TD task was over, patients were administered the BDI-II.

#### Data Analysis

For each task, the rate at which the subjective value of a reward decays with delay (TD rate) was assessed through two indices: the TD parameter (*k*; Mazur, 1987; Rachlin et al., 1991; Green and Myerson, 2004), and the area under the empirical discounting curve (AUC; Myerson et al., 2001).

##### Estimation of k

The hyperbolic function *SV* = 1/(1 + *kD*), where *SV* = subjective value (expressed as a fraction of the delayed amount), and *D* = delay between the two options (in days), was fit to the data to determine the *k* constant of the best fitting TD function, using a nonlinear, least squares algorithm (as implemented in StatisticaStatsoft^®^). The larger the value of *k*, the steeper the discounting function, the more participants were inclined to choose smaller sooner rewards over larger later rewards. Subjective preferences were well characterized by hyperbolic functions, as indexed by high *R*^2^ across participant groups and temporal conditions (*R*^2^ > 0.64 in all cases). For comparison purposes, we also assessed the fits to the data of an exponential discounting model. For each TD task, the exponential function *SV* = exp^−*kD*^ was fit to the data to determine the *k* constant of the best fitting function. The hyperbolic function proved to fit the data better than the exponential functions across participant groups and temporal conditions. We entered *R*^2^ scores as the dependent variable in an analysis of variance (ANOVA) with Group [Insular patients, Non-insular patients, healthy controls (HC)] as a between-subject factor, and Model (hyperbolic, exponential) and Temporal condition (Now, Not-now) as within-subject factors. There was a significant effect of Model (*F*_(1,86)_ = 16.0, *p* = 0.0001). *Post hoc* comparisons, performed with the Fisher test, showed that *R*^2^ values were significantly higher for the hyperbolic than the exponential model (0.72 vs. 0.66, *p* = 0.000001). No other effects were significant (*p* > 0.19 in all cases). Given the superiority of the hyperbolic over the exponential model in describing TD behavior, hyperbolic *k* values were adopted as measures of TD. The hyperbolic *k* constants were normally distributed after log-transformation (Kolmogorov-Smirnov *d* < 0.09, *p* > 0.20 in all cases), and therefore comparisons were performed using parametric statistical tests.

##### Estimation of AUC

Although hyperbolic functions captured participants’ TD behavior relatively well, we also obtained AUC as an additional index of TD rate that, unlike *k*, does not depend on theoretical models regarding the shape of the discounting function (see Myerson et al., 2001). After delays and subjective values were normalized, delays were expressed as a proportion of the maximum delay, and subjective values were expressed as a proportion of the greater amount (40 €). Delays and subjective values were then plotted as *x* and *y* coordinates, respectively, to construct a discounting curve. Vertical lines were drawn from each *x* value to the curve, subdividing the area under the curve into a series of trapezoids. The area of each trapezoid was calculated as (*x_2_ − x_1_*)(*y_1_ + y_2_*)/2, where *x_1_* and *x_2_* are successive delays, and *y_1_* and *y_2_* are the subjective values associated with these delays (Myerson et al., 2001). The AUC is the sum of the areas of all the trapezoids and varies between 0 and 1. The smaller the AUC, the steeper TD, the more participants were inclined to choose smaller sooner rewards over larger later rewards. The AUC scores were normally distributed (Kolmogorov-Smirnov *d* < 0.10, *p* > 0.20 in all cases), and therefore comparisons were performed using parametric statistical tests.

#### Voxel-Based Lesion-Symptom Mapping (VLSM)

Standard groupwise comparisons were supplemented with a Voxel-based Lesion-Symptom Mapping (VLSM) analysis oriented at investigating the relation between brain damage and behavior on a voxel-by-voxel basis. VLSM allows lesion-behavior associations to be tested without assigning patients to arbitrary groups. In this method, a behavioral measure is entered as the dependent variable, and the lesion status of each voxel (lesioned or not) is the independent variable. Then, for each voxel, statistical comparisons are made between the performance of subjects with vs. without lesions affecting that voxel. The output is a statistical map indicating voxels associated with poor performance when lesioned (Bates et al., 2003).

We entered patients’ TD scores (*k* and AUC) in the Non-Parametric Mapping software (NPM; Rorden et al., 2007), separately for the Now and Not-now condition. The software compares performance of patients with vs. without damage at each voxel using the nonparametric Brunner-Munzel (BM) rank-order test (Brunner and Munzel, 2000). The higher the resulting statistical output (*Z* value) relative to voxels in a given area, the stronger the association between damage in that area and impaired performance. Only voxels affected in at least 20% of cases were included for the analysis. The alpha level of significance was set at *p* < 0.05, corrected for False Discovery Rate (FTD; Nichols and Hayasaka, 2003), and an extent threshold of 50 voxels per cluster was adopted (see also Gläscher et al., 2010).

### Results

#### Temporal Discounting

**Figure 4:**
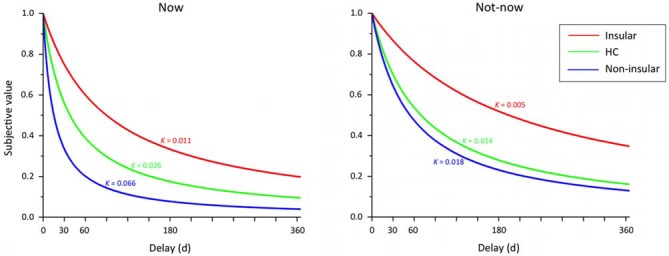
**Temporal discounting (TD) functions by participant group (Insular = patients with lesions in the insular cortex; Non-insular = patients with lesions outside the insular cortex; HC = healthy controls) and type of temporal condition.** The hyperbolic curves describe the discounting of subjective value (expressed as a proportion of the delayed amount) as a function of time (days). The discounting parameter *k* reflects the geometric mean of the group.

**Figure 5:**
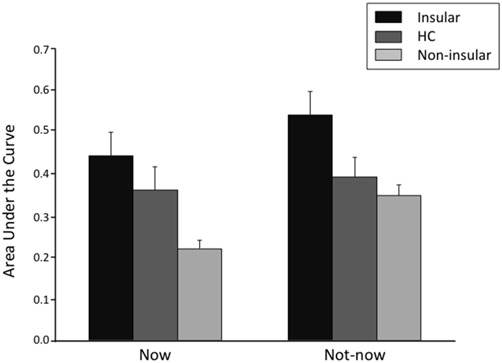
**Area under the empirical discounting curve (AUC) by participant group (Insular = patients with lesions in the insular cortex; Non-insular = patients with lesions outside the insular cortex; HC = healthy controls) and type of temporal condition.** The error bars indicate the standard error of the mean.

Figure 4 shows TD curves by participant group and temporal condition. The *k* value for each curve reflects the geometric mean of the group, and thus provides a better measure of central tendency for positively skewed metrics—such as TD rates—than does the arithmetic mean. Figure 5 shows the AUC by participant group and temporal condition. As is evident from the figures, Insular patients discounted future rewards less steeply (Insular Patients, Now: geometric mean’ *k* = 0.011, *SD* = 0.03; Not-now: geometric mean’ *k* = 0.005, *SD* = 0.01) than the control groups (Non-insular patients, Now: geometric mean’ *k* = 0.066, *SD* = 0.13; Not-now: geometric mean’ *k* = 0.018, *SD* = 0.03; HC, Now: geometric mean’ *k* = 0.026, *SD* = 0.12; Not-now: geometric mean’ *k* = 0.014, *SD* = 0.27). For example, the delay at which 40 € decreased to 50% of their original value (so that they were worth 20 € now) was about 38 days for normal controls, but ~90 days for Insular patients. These impressions were confirmed by ANOVA analyses.

##### k

An ANOVA on log-transformed *k* values with Group (Insular patients, Non-insular patients, HC) as a between-subject factor, and Temporal condition (Now, Not-now) as a within-subject factor yielded a significant effect of Group (*F*_(2,86)_ = 6.01, *p* = 0.003). *Post hoc* comparisons, performed with the Fisher test, showed that TD was less steep in Insular patients compared to Non-insular patients (−2.12 vs. −1.46; *p* = 0.001) and HC (−2.12 vs. −1.71; *p* = 0.008), whereas no significant difference was detected between Non-insular patients and HC (*p* = 0.09). Moreover, there was a significant effect of Temporal condition (*F*_(1,86)_ = 22.44, *p* = 0.000008), indicating that TD was generally steeper in the Now compared to the Not-now condition (−1.57 vs. −1.88; *p* = 0.000002). There was no significant Group × Temporal condition interaction (*F*_(2,86)_ = 1.39, *p* = 0.25).

##### AUC

Similar results were obtained using AUC as the dependent variable. An ANOVA on AUC scores with Group and Temporal condition as factors yielded a significant effect of Group (*F*_(2,86)_ = 4.79, *p* = 0.01). Fisher *post hoc* comparisons showed that AUC was larger (i.e., TD was slower) in Insular patients compared to Non-insular patients (0.49 vs. 0.28; *p* = 0.003) and HC (0.49 vs. 0.38; *p* = 0.03), with no difference between the latter two groups (*p* = 0.07). Again, there was a significant effect of Temporal condition (*F*_(1,86)_ = 14.91, *p* = 0.0002), indicating smaller AUCs in the Now condition than in the Not-now condition (0.35 vs. 0.40; *p* = 0.002), but no significant Group × Temporal condition interaction (*F*_(2,86)_ = 2.69, *p* = 0.07).

##### Control analysis

Given that there were four Non-insular patients out of thirteen, whose lesion involved the superior frontal cortex, and that lesions in the frontal cortex can result in increased impulsivity (Floden and Stuss, 2006; Olson et al., 2009), to exclude that Non-insular patients performance was driven by these patients we reran the main analyses excluding them from the sample. We confirmed our results. In particular, in the ANOVA on log-transformed *k* values a significant effect of Group (*F*_(2,86)_ = 4.73, *p* = 0.01) emerged, such that TD was less steep in Insular patients compared to Non-insular patients (−2.12 vs. −1.49; *p* = 0.005) and HC (−2.12 vs. −1.71; *p* = 0.01), whereas no significant difference was detected between Non-insular patients and HC (*p* = 0.22). Moreover, there was a significant effect of Temporal condition (*F*_(1,86)_ = 16.11, *p* = 0.0001), indicating that TD was generally steeper in the Now compared to the Not-now condition (−1.59 vs. −1.89; *p* = 0.00001). There was no significant Group × Temporal condition interaction (*p* = 0.53). The same applies for the analysis on AUCs: there was a significant effect of Group (*F*_(2,86)_ = 3.34, *p* = 0.04), such that TD was less steep in Insular patients compared to Non-insular patients (0.49 vs. 0.31; *p* = 0.02) and HC (0.49 vs. 0.37; *p* = 0.03), whereas no significant difference was detected between Non-insular patients and HC (*p* = 0.26). Moreover, there was a significant effect of Temporal condition (*F*_(1,86)_ = 12.05, *p* = 0.001), indicating that TD was generally steeper in the Now compared to the Not-now condition (0.36 vs. 0.41; *p* = 0.006).

#### Choice Consistency

Further analyses were run in order to exclude that our results were due to idiosyncratic TD behavior or inconsistent choices on the Insular patients’ part. First, no participant in any group followed a response heuristic, such as always selecting the larger-delayed amount or the smaller-immediate amount across delay and temporal conditions.

Turning to inconsistent choices, TD behavior should result, by definition, in a monotonic decrease of the subjective value of the future outcome with delay (Johnson and Bickel, 2008). Thus, if *R1* is the subjective value of a reward *R* delivered at delay *t1*, *R2* is the subjective value of *R* delivered at delay *t2*, and *t2* > *t1*, then it is expected that *R2* < *R1*. Therefore, subjects exhibit inconsistent preference when the subjective value of the future outcome at a given delay is greater than that at the preceding delay, i.e., *R2* > *R1* (Johnson and Bickel, 2008). To allow variability in the data, we considered as indicative of inconsistent preferences only those data points in which the subjective value of a reward overcame that at the preceding delay by a value of >10% of the future outcome, i.e., *R2* > *R1* + *R*/10, as recommended by Johnson and Bickel (2008). An ANOVA on the number of inconsistent preferences (in which the subjective value of a reward overcame that at the preceding delay by a value of >10% of the future outcome) with Group (Insular, Non-insular, HC) as a between-subject factor, and Temporal condition (Now, Not-now) as a within-subject factor yielded no significant effect of Group (*F*_(2,86)_ = 0.58, *p* = 0.56), no significant effect of Temporal condition (*F*_(1,86)_ = 0.05, *p* = 0.82), and no significant Group × Temporal condition interaction (*F*_(2,86)_ = 1.77, *p* = 0.18). These results indicate clearly that all groups made a comparable number of inconsistencies in both temporal conditions (mean number of inconsistent preferences for the Now condition: Insular, 0.60; Non-insular, 0.90; HC, 0.70; Not-now condition: Insular, 1.0; Non-insular, 0.70; HC, 0.60). This held even if all deviations from a monotonically decreasing function were counted as inconsistent preferences, regardless of their magnitude, i.e., *R2* > *R1* (all *p*’s > 0.20).

Together, the findings that lesion to the insular cortex did not result in changes to the shape of the discounting function (aside from its steepness), response heuristics or inconsistent preferences, strongly suggest that Insular patients’ behavior was indeed reflective of decreased TD, and not poor task comprehension or idiosyncratic preferences.

#### VLSM

**Figure 6:**
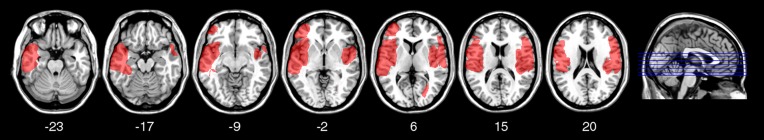
**Statistical power map.** Map showing the voxels (in red) where there is sufficient statistical power to detect an effect in this group of patients, overlaid on the MNI brain. In each axial slice, the left hemisphere is on the left side. *Z*-coordinates of each axial slice are given.

**Figure 7:**
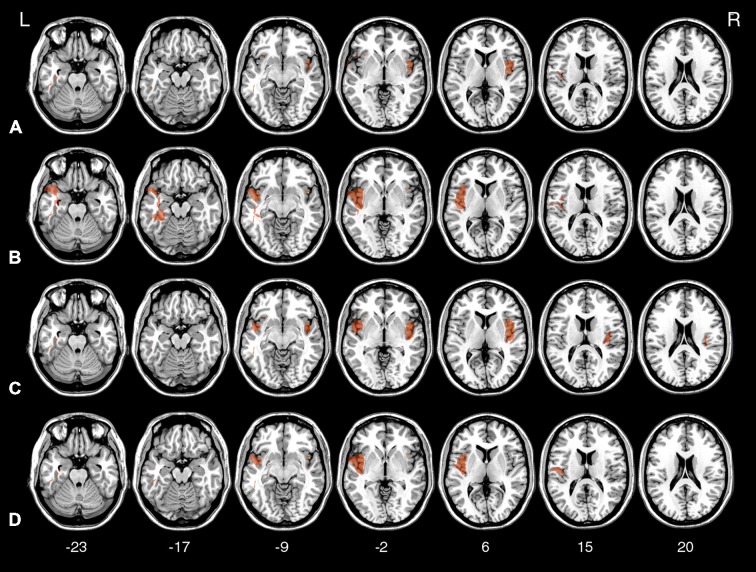
**Voxel-based lesion-symptom mapping (VLSM) statistical map computed for *k* in the Now condition (A), *k* in the Not-now condition (B), AUC in the Now condition (C), and AUC in the Not-now condition (D), thresholded at *p* < 0.05, FDR-corrected, and shown on representative axial slices of the MNI brain.** *Z*-coordinates of each axial slice are given.

To investigate the relation between TD deficits and specific brain lesions, we performed a VLSM analysis. The VLSM analysis related patients’ *k* rates and AUC values for the Now and Not-now conditions (in separate analyses) to their brain lesions. Figure 6 shows the statistical power map, indicating the voxels where we had adequate power to detect effects with a 5% FDR threshold (Rorden et al., 2007; Gläscher et al., 2010). The brain regions associated with reduced TD, along with the coordinates of their center of mass, based on the MNI brain atlas, are listed in Table 2 and shown in Figure 7.

**Table 2:** **VLSM results**.

	Hemisphere	*x*	*y*	*z*	Cluster size (voxel)	BA	Max
***k***
**Now condition**
Insula	Right	44	8	−10	4596	–	2.67
Insula	Left	−37	6	9	295	–	2.30
Temporal superior	Left	−63	3	−3	128	48	2.09
Temporal medial	Left	−51	−34	−15	97	20	2.05
Temporal inferior	Left	−50	−35	−24	276	20	2.04
Insula	Left	−34	21	6	333	48	1.96
Rolandic operculum	Left	−40	−19	14	61	48	1.93
Insula	Left	−36	18	−9	133	47	1.80
***k***
**Not-now condition**
Insula	Right	41	8	−10	122	48	2.67
Fusiform gyrus	Left	−38	−15	−22	28021	20	2.45
Temporal superior	Left	−55	−14	13	76	48	1.69
**AUC**
**Now condition**
Insula	Right	43	8	−10	9733	48	−2.67
Temporal inferior	Left	−50	−35	−24	276	20	−2.10
Temporal medial	Left	−51	−34	−15	97	20	−2.10
Insula	Left	−36	18	−9	133	47	−1.79
**AUC**
**Not-now condition**
Insula	Right	44	8	−10	247	–	−2.48
Temporal superior	Left	−57	2	1	11869	48	−2.37
Fusiform gyrus	Left	−38	−23	−25	838	20	−2.14

*Coordinates of the regions associated with reduced TD in the VLSM analysis on both k and AUC values, in MNI space. Region labels are taken from the AAL. BA, Brodmann area; Max, maximum BM Z statistics obtained for each cluster. Z scores are significant at a threshold of p < 0.05, FDR-corrected*.

As indicated in Table 2, the largest clusters and the highest *Z*-values, for both the analysis on *k* and AUC values, were located in the insula. In both cases, in the Now condition, the highest concentration of significant voxels was in the right insula. A second distinct cluster of significant voxels was located in the left insula. There were other, smaller and less reliable clusters of voxels associated with reduced TD, including more anterior portions of the left insula and regions in the lateral temporal lobe, as listed in Table 2. As well, in the Not-now conditions, for both the analysis on *k* and AUC values, the voxels with the highest *Z*-values were again located in the right insula, and in an extended cluster in the left hemisphere—with peak in the left fusiform gyrus for the analysis on *k*, and in the superior temporal lobe for the analysis on AUC—that also included the insula. Again, smaller and less significant clusters were detected in the left temporal lobe.

Thus, the VLSM results confirm that the abnormal TD behavior of Insular patients was mainly driven by damage to the insula. As anticipated, the VLSM analysis also revealed other regions, mainly in the temporal lobe, that were also related to TD behavior, although less reliably. The emergence of these additional regions in the VLSM analysis is difficult to interpret, primarily because we have no hypotheses on their putative role on TD. Additionally, damage in some of those regions (operculum, superior temporal pole) correlates with insula damage in this data set.

#### Self Report of Depression

Insular patients’ self-reports did not evince significantly higher levels of depression at the BDI-II than did those of Non-insular patients (*F*_(1,23)_ = 0.27; *p* = 0.61; see Table 1). Separate analysis on the scores from the affective and somatic subscales of the BDI-II also failed to yield statistically significant results (*p* > 0.54 in both cases). Thus, any difference in the discounting behavior of Insular and Non-insular patients cannot be explained by increased level of depression in patients with insular cortex lesion.

## Experiment 2

Two corollary investigations were conducted. To provide support to our hypothesis that decreased TD in Insular patients may depend on reduced emotional responses during decision-making, we had participants rate the valence and the arousal evoked by positive, negative, and neutral stimuli (Emotion task). Moreover, given that some studies have detected activity in the insula during tasks requiring time perception (e.g., Craig, 2009; Wittmann et al., 2010; Schirmer, 2011), we ran an additional task aimed at excluding time estimation deficits in Insular patients (Time estimation task).

### Materials and Methods

#### Participants

Participants included three patients from the Insular group of the main experiment—recruited about 24 months after the primary study—and one additional patient who had not taken part in the main experiment (Insular patients; 3 males; right hemisphere: 2 cases; mean age = 44.2; *SD* = 19.0; mean education = 12; *SD* = 1.5), and 12 healthy individuals matched to the patients on age, education, and gender balance (3 females; mean age = 49.3; *SD* = 12.5; mean education = 15.1; *SD* = 4.1; all *p*’s > 0.14). The new Insular patient had lesion restricted to the anterior portion of the right insula as well as to the right frontal operculum. His cognitive functioning was in the normal range, as documented by the DS forward test (corrected score = 5.75), and the CRM (corrected score = 49.25; Spinnler and Tognoni, 1987).

#### Emotion Task

Twenty-five images were selected from the International Affective Picture System (IAPS; Lang et al., 1999). Ten were positively valenced (e.g., people smiling, relaxing places), 10 were negatively valenced (e.g., people crying, scenes with weapons), and five had a neutral content (e.g., objects, people with a neutral expression). Mean valence levels from Lang et al. (1999) were 7.05, 3.32, and 5.08 for the positive, negative, and neutral set, respectively, whereas mean arousal levels for the selected pictures were 5.01, 4.74, and 4.31, and was not significantly different across sets of pictures (*p* > 0.64). Stimuli were selected to sample both social and nonsocial context.

Each picture was presented on a computer screen for 10,000 ms. Participants rated each picture using the Self-Assessment Manikin (Lang et al., 1999), a 9-point scale allowing self-report of valence, ranging from 1 (happy) to 9 (sad), and arousal, ranging from 1 (activated) to 9 (relaxed).

#### Time Estimation Task

We used a time estimation task adapted from Livesey et al. (2007). In each trial, two black squares (20 mm × 20 mm) appeared consecutively on a white background, centrally, with a 700 ms blank screen gap. One of the two stimuli remained on the screen 1000 ms longer than the other. In 16 trials, one stimulus appeared for 1000 ms and the other for 2000 ms. In the other 16 trials, one stimulus appeared for 2000 ms and the other for 3000 ms. Two catch trials were included, in which one stimulus remained on the screen for 1000 ms and the other for 3000 ms. The order of shorter and longer stimuli was counterbalanced. After the second stimulus, a yellow dot appeared centrally, which required participants to indicate whether the first or the second stimulus had lasted longer, using one of two buttons. There was no time limit for responding. After an inter-trial interval of 1500 ms, indicated by a central fixation cross, a new trial began.

### Results

#### Emotion Task

Arousal scores revealed that insular patients felt less activated at the view of positive pictures than HC (Insular: *M* = 5.37, *SD* = 0.96; HC: *M* = 3.98, *SD* = 0.82), Mann-Whitney *U* = 5.5, *p* = 0.02. There was no difference between groups in arousal scores for negative and neutral pictures, as well as in valence ratings across all picture sets (all *p*’s > 0.33).

#### Time Estimation Task

No subject failed at catch trials. There was no difference in the frequency of correct responses between groups (30 vs. 31), Mann-Whitney *U* = 21, *p* = 0.70.

## Discussion

The present study investigated the role of the insular cortex in intertemporal choice. Patients with lesions involving the insular cortex, and healthy as well as brain-damaged controls made choices between smaller-sooner and larger-later amounts of money. Two temporal conditions were tested: in one, the earlier quantity of money was delivered immediately, whereas in the other it was delayed by 60 days. Lesion to the insula significantly reduced TD of future rewards: Insular patients behaved more patiently than control participants, being less sensitive to sooner rewards. This finding held in both the Now and Not-now condition. Notably, all participants, including Insular patients, showed a significant decreased TD in the Not-now compared to the Now condition: They behaved more impatiently when the sooner option was available immediately than when both options were delayed in time, replicating previous findings in healthy individuals (Frederick et al., 2002; McClure et al., 2004; Green et al., 2005; Figner et al., 2010; but see Kable and Glimcher, 2010).

It is important to note that reduced TD in Insular patients is not likely to be attributable to a general effect of brain damage, because Non-insular patients showed normal TD. Moreover, in a previous report, we showed that patients with lesion to the mOFC consistently prefer smaller-sooner over larger-later reward (Sellitto et al., 2010), a behavior that is opposite to the one exhibited by Insular patients. It is also unlikely that our findings were due to a general insensitivity to reward, or blatant problems at estimating the passage of time in Insular patients. First, all patients consistently chose the larger reward in the control conditions of the TD task. Second, Insular patients behaved more impulsively in the Now compared to the Not-now condition, and this tendency was as pronounced in Insular patients as it was in the controls, indicating that Insular patients were generally able to represent the passage of time. Moreover, a corollary investigation task found no blatant deficits in time estimation in Insular patients compared to healthy individuals.

Thus, our findings indicate that the insula plays a crucial role during intertemporal choice, contributing to shape TD behavior. Several fMRI studies detected activity in the insula modulates according to the time of availability of edible and monetary outcomes (Tanaka et al., 2004; McClure et al., 2007; Wittmann et al., 2007, 2010; Claus et al., 2011; Liu and Feng, 2012; Luo et al., 2012). Increased insula activity, however, has been reported in association with both delayed (Wittmann et al., 2007; Claus et al., 2011; Kayser et al., 2012) and immediate rewards (Tanaka et al., 2004; McClure et al., 2007; Wittmann et al., 2010), and therefore its role in TD was unclear. The fact that Insular patients behaved more patiently than both healthy and brain-damaged controls, forgoing sooner rewards to receive later ones, indicates that the insula is necessary during intertemporal choice. This was confirmed by a VLSM analysis showing that the association between lesions in the insula and abnormal TD behavior was statistically significant. In particular, the less steep TD evinced by Insular patients (compared to controls) suggests that the insula is normally implicated in upregulating the incentive value of relatively sooner reward options. How might the insula accomplish such a role?

We argue that, during decision-making, the insula may anticipate the emotional/bodily effects of different choice options, contributing to their incentive value (Barrett et al., 2007). In particular, in a TD task involving receiving rewards at different delays, the insula may signal the urge to obtain a reward as soon as possible. Damage to the insula, therefore, would diminish the motivation to obtain a reward soon, allowing patients to wait for larger-later outcomes, thus reducing TD. This interpretation makes contact with extensive evidence implicating the insula in craving associated with cigarette and alcohol addiction, a paradigmatic condition of capitulation to immediate rewards despite bad long-term consequences (e.g., Wang et al., 2007; Hoffman et al., 2008; Paulus et al., 2009; Kenny, 2011; Claus et al., 2011; Kang et al., 2012; Sutherland et al., 2012; Vaidya et al., 2012). Crucially, lesion to the insula disrupts addiction to smoking (Naqvi et al., 2007), as if the patients’ “body forgot the urge to smoke” (p. 534), confirming that insula activity promotes courses of action directed at satisfying current needs. Another clinical population exhibiting an apparent ability to resist current temptations is that of individuals with anorexia nervosa, who sustain self-denial of food (Kaye et al., 2009). Anorexic patients, too, show reduced TD (Steinglass et al., 2012), and functional abnormalities in the insula (Frank et al., 2012; Gaudio and Quattrocchi, 2012).

One may argue that, instead of reducing the urge for reward, damage to the insula reduced the feeling of uncertainty related to waiting for delayed rewards (Tom et al., 2007; Clark et al., 2008; Christopoulos et al., 2009). Even though intertemporal choices are not typically designed as risky choices, delay may influence choice via the perceived risk of loss inherently associated with waiting (Paulus et al., 2003; Kuhnen and Knutson, 2005; Knutson et al., 2007). Our TD task does not allow distinguishing whether damage to the insula reduced the urge to obtain something positive (a reward soon), or to avoid something negative (the fear of loss related to waiting for a later reward). Although gathered from a small sample, however, the finding that in the control Emotion task Insular patients showed decreased arousal for positive (but not negative or neutral) pictures makes us lean towards the former interpretation. Insular patients may be not sufficiently activated in response to positive outcomes, such as monetary rewards, to seek them as soon as possible.

Notably, in a previous report, we showed that mOFC-lesioned patients consistently prefer smaller-sooner over larger-later reward (Sellitto et al., 2010), a behavior that is opposite to the one exhibited by Insular patients in the present study. The two studies are not strictly comparable due to methodological differences between the tasks used (including different time frames, procedures, and types of reward). However, we would like to offer an interpretation of the mechanistic interplay between OFC and the insula during intertemporal choice. It has been proposed that mOFC, along with the adjacent medial prefrontal cortex and the ventral striatum, takes part in a system representing the subjective value of both immediate and delayed outcomes, under the top-down control by lateral prefrontal cortex (Kable and Glimcher, 2007, 2010; Christakou et al., 2009, 2011; Hare et al., 2009; Figner et al., 2010). Within this network, mOFC and adjacent medial prefrontal regions are thought to weight the long-term outcome of choices (Schoenbaum et al., 2009; Sellitto et al., 2010), possibly through prospection (Ciaramelli and di Pellegrino, 2011), whereas the ventral striatum may convey signals of immediate pain or pleasure (Bechara and Damasio, 2005; Kringelbach, 2005). Insula possesses connections with both the ventromedial prefrontal cortex and the ventral striatum (Reynolds and Zahm, 2005). Therefore, during intertemporal choice, it may relay interoceptive inputs about need states to both systems, determining the strength with which individuals will pursue a reward option or the other (Weller et al., 2009; Droutman et al., 2015). According to this model, damage to mOFC would cause a problem valuing future outcomes, leading to steep TD, whereas damage to the insula would lead to “emotionally blunt” intertemporal choices, which would tend to be based on a heuristic of quantity, leading to reduced TD. This is, indeed, what we have observed in brain-damaged patients (Sellitto et al., 2010; this study).

Our results may have clinical implications. Several studies have documented dysfunctional activation of the insula and altered connectivity between the insula and other reward-related brain regions in individuals with addiction, who also typically show steep TD (e.g., Garavan et al., 2000; Li et al., 2000; Daglish et al., 2003; Garavan, 2010; Droutman et al., 2015). Sutherland et al. (2012) have proposed that functional alterations in the insula and the addiction-related circuit may be even considered as a biomarker of addiction treatments efficacy. Our study concur with previous evidence (e.g., Naqvi et al., 2007) in suggesting that treatments targeting processing in the insula may be effective in reducing some aspects of addiction.

In conclusion, we have shown that damage to the insula causes increased willingness to wait in intertemporal choice. While far-sighted decision-making has obvious advantages, in many situations in life it is important, and preferred, to pursue current opportunities instead of waiting for potential future ones, as captured in the popular saying “every missed chance is lost forever”. The present results point to the insula as crucial to pursue current rewards, and take chances as soon as possible, favoring action over prospection.

## Author Contributions

MS, EC, and GdP conceived the design of the work; MS, EC, and GdP collected, analyzed and interpreted the data; MS, EC, and GdP wrote the manuscript and all authors approved the final version.

## Conflict of Interest Statement

The authors declare that the research was conducted in the absence of any commercial or financial relationships that could be construed as a potential conflict of interest.

## Footnotes

^1^http://www.bic.mni.mcgill.ca/ServicesAtlases/ICBM152Lin
